# Interactions With Relatives of Critically Ill Non‐Western Immigrants: Do We Have a Blind Spot?

**DOI:** 10.1111/aas.70082

**Published:** 2025-06-18

**Authors:** Mitti Blakoe, Christian Sylvest Meyhoff, Ditte Strange

**Affiliations:** ^1^ Department of Anaesthesia and Intensive Care, Bispebjerg and Frederiksberg Hospital University of Copenhagen Copenhagen Denmark; ^2^ Department of Anesthesia and Intensive Care Zealand University Hospital Roskilde Denmark

Communication between ICU healthcare professionals and relatives can break down for various reasons. One possible reason is cultural dissonance, where professionals may not understand the behaviors and wishes of relatives of critically ill non‐Western patients, and even the best communicators face challenges without cultural understanding [1, 2].

Culture encompasses the self‐explanatory behaviors and values shared by a community or group. This principle also applies to the culture in the Scandinavian healthcare system, where professionals adhere to established norms related to their roles, as well as those of patients and relatives during a hospital stay [2]. Historically, patients and families have complied with these implicit norms of hospital etiquette. However, in recent decades, the population of immigrants from non‐Western countries in Scandinavia has risen to approximately 10% [3], introducing diverse norms for hospital etiquette that may conflict with established practices. This is especially evident in the context of critical illness [4, 5]. For example, non‐Western immigrants may use religious coping more extensively by viewing life and death as being in God's hands rather than solely under the control of healthcare professionals [6, 7]. Such cultural differences may lead to misunderstandings and potential conflicts, especially in end‐of‐life situations, where a medical decision to withdraw treatment may contradict religious or cultural beliefs. Additionally, some cultures emphasize the family and friends' obligation to visit and care for their loved ones, which potentially creates tension when numerous relatives wish to visit patients in a hospital that promotes a visitor limitation policy to ensure peace and privacy among co‐patients [6]. These and other potential conflicts may be rooted in different cultural norms, the polarities of which are illustrated in Figure 1.

**FIGURE 1:**
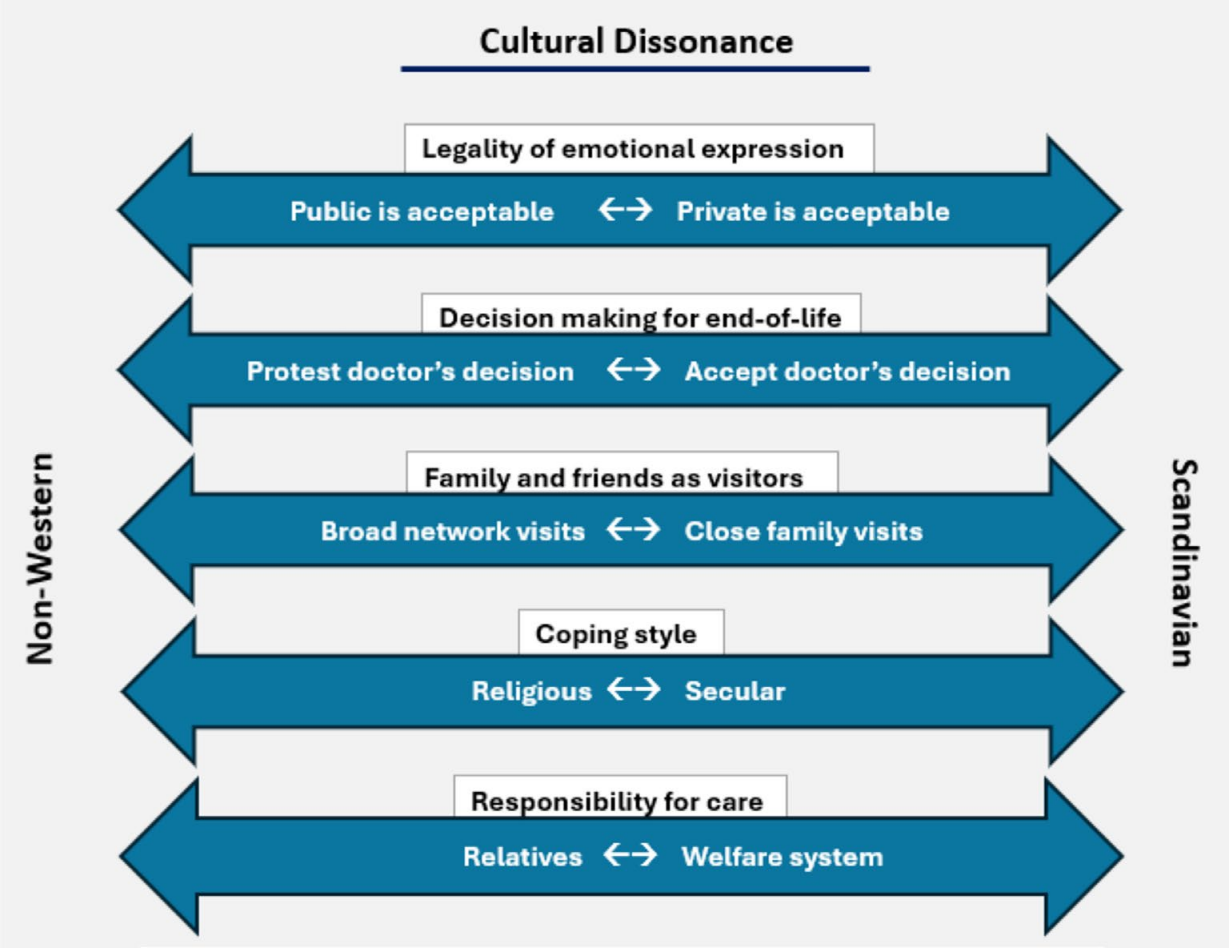
Examples of cultural dissonance between non‐Western relatives' cultural norms and the Scandinavian health care system's cultural norms.

Fortunately, clinical practice is more nuanced and fragmented than illustrated in Figure 1. It is, however, not uncommon to experience some level of cultural dissonance, which can have consequences in ICUs and other critical care departments. Evidence suggests that relatives may perceive it as discrimination, which can negatively affect their future help‐seeking and health‐related behaviors [8, 9]. Healthcare reforms in Scandinavian countries aim to enhance the involvement of relatives during hospitalization [10]. Healthcare professionals will therefore have an obligation to mediate consonance in the interaction, regardless of the patients' and relatives' cultural background.

In recent years, successful cross‐cultural initiatives have been established in the ICU at Bispebjerg Hospital in Copenhagen, Denmark. These initiatives include all doctors and nurses in our ICU to: (1) Allow large groups of relatives into the ICU area and around the ICU bed at the end of life; (2) Recognize grief, even when it is loud; (3) Understand that when family members hesitate to end a patient's life, it is often an expression of love and protection; (4) Accept that more time may be needed before transitioning to the palliative phase. These initiatives have been implemented through internal education, which has reshaped employees' expectations regarding the norms for relative behaviors at the ICU and has resulted in a much more confident and comfortable environment, where relatives often express deep gratitude for how we manage the critical situation of illness.

Several blind spots do, however, remain in the healthcare system [11]. Most informational materials exist only in the native language, and e‐health initiatives often overlook linguistic and cultural barriers, increasing the risk of misunderstandings and medical errors [12]. Non‐native speakers are also frequently excluded from interventional studies and surveys, leading to research findings that reflect Scandinavian or Western values, which may not suit non‐Western residents in our countries. Excluding non‐Western immigrants and their relatives from these studies risks depriving them of future healthcare opportunities; thus, researchers may unintentionally contribute to health inequality.

If we want to address and reduce this cultural dissonance in the context of critical illness and uphold the principles of our healthcare reforms, we must initiate and support cross‐cultural local initiatives in ICUs across Scandinavia. Furthermore, even with potential language obstacles, we must make a determined effort to involve non‐Western immigrants in clinical studies to mitigate health inequalities for these patients and their relatives.

The experiences and suggestions in this editorial are our modest contribution to highlighting the blind spot.

## Author Contributions

All authors participated in conceptualizing the manuscript. M.B. wrote the initial draft, and all authors revised it.

## Conflicts of Interest

None related to the present manuscript, but Christian Sylvest Meyhoff has founded a spin‐out company, WARD24/7 ApS, with the aim of pursuing the regulatory and commercial activities of the WARD‐project (Wireless Assessment of Respiratory and circulatory Distress, a project developing a clinical support system for continuous wireless monitoring of vital signs). WARD24/7 ApS has obtained license agreement for any WARD‐project software and patents. One patent has been filed: “Wireless Assessment of Respiratory and circulatory Distress (WARD), EP 21184712.4 and EP 21205557.8.”

## Data Availability

Data sharing is not applicable to this article as no new data were created or analyzed in this study.
